# Achieving Optimal Transfection Conditions in Chicken Primordial Germ Cells Under Feeder- and Serum-Free Medium

**DOI:** 10.3390/ani15040590

**Published:** 2025-02-18

**Authors:** Zhifeng Zhao, Xian Zou, Ying Zhu, Yanhua He, Endashaw Jebessa, Jiannan Zhang, Jian Ji, Peng Chen, Chenglong Luo

**Affiliations:** 1 State Key Laboratory of Swine and Poultry Breeding Industry, Guangdong Key Laboratory of Animal Breeding and Nutrition, Institute of Animal Science, Guangdong Academy of Agricultural Sciences, Guangzhou 510640, China; 2 Key Laboratory of Bio-Resources and Eco-Environment of Ministry of Education, College of Life Sciences, Sichuan University, Chengdu 610064, China

**Keywords:** primordial germ cell, feeder free, electroporation, transfection efficiency, CRISPR/Cas 9

## Abstract

The successful application of primordial germ cells (PGCs) is an ideal method for generating gene-edited birds. However, barriers to efficient DNA transfection in PGCs lead to low transfection efficiency, limiting the generation of genetically modified chickens. The current study utilized chemical transfection and electroporation methods to determine the optimal transfection conditions for the PGC line under feeder- and serum-free medium. Among the tested methods, the Lonza electroporation system exhibited the highest transduction efficiency, with a previously unreported rate of 71.13 ± 1.26%. Optimal transfection conditions were achieved using 4 µg of DNA and 100 µL of Entranster^TM^-E in 1 × 10^6^ PGCs. Furthermore, the optimal electroporation conditions resulted in low cell death and normal expression of pluripotency-related genes, highlighting the low cytotoxicity. The resulting electroporation models were then used to deliver the enhanced green fluorescent protein (*EGFP*) gene to the Z chromosome with a Cas9-gRNA plasmid, achieving a 7-day insertion efficiency of 14.63 ± 1.07%. Our study highlights the vast potential of electroporation technology for the transfection of PGCs.

## Simple Summary

Producing genetically modified birds is crucial for agricultural and medical research, while current methods face challenges in transfection efficiency of primordial germ cells (PGCs). The current study aimed to improve the process of delivering foreign genes into PGCs under feeder- and serum-free conditions. We found that the electroporation of Lonza system achieved the highest transfection efficiency (over 70%). This method also maintained normal cell growth and function. Using this optimal approach, we successfully inserted a fluorescent marker gene into a specific location on a chicken Z chromosome, achieving a 14.63 ± 1.07% success rate. These results demonstrate that electroporation is a safe and efficient tool for modifying the genome of PGCs. The research optimized the efficiency of gene delivery in PGC, paving the way for studying gene expression and integrating gene editing technologies into PGCs.

## 1. Introduction

Transfection is a fundamental technique for studying gene expression and integrating gene editing technologies into cells. Commonly used transfection methods include lipofectamine-mediated transfection, viral infection, and electroporation, each with unique advantages and limitations. Lipofectamine-mediated transfection is one of the most commonly used and straightforward methods for cell line transfection. This method involves the formation of complexes between transfection reagents and nucleic acids, which are delivered into cells via membrane fusion or endocytosis [1]. However, lipofectamine transfection of some primary cells using lipofectamine is still inefficient, especially in primordial germ cells (PGCs) [2]. Viral transfection utilizes the natural ability of viruses to infect cells and deliver exogenous genes into host cells. However, the biosafety concerns and the limitation in the cargo capacity of exogenous nucleic acids restrict the widespread application of viral transfection [3]. On the other hand, electroporation utilizes electrical pulses to temporarily disrupt the cell membrane, allowing the entry of molecules such as DNA or RNA [4,5]. This method is theoretically suitable for various cell types, including hard-to-transfect cells like stem cells. Electroporation is simple, rapid, and scalable, while high-voltage pulses may damage or kill cells. Therefore, optimizing electrical pulse parameters is critical to balancing transfection efficiency and cell viability. These transfection methods are indispensable tools in gene editing and functional genomics, especially in primary cell and stem cell research, which are pivotal for advancing animal genetics, breeding, and reproductive biology.

PGCs are ideal models for conserving avian genetic resources and studying avian germline development [6]. As the first population of germ cells during embryonic development, PGCs can be purified, cryopreserved, and revived for ex situ conservation and in vitro preservation of poultry genetic resources [7,8,9,10]. Rare poultry breeds derived from donor PGCs can be successfully regenerated by transplanting cryopreserved PGCs into embryos [11]. Moreover, avian PGCs have the unique ability to proliferate in vitro and undergo genetic modifications, making them a valuable tool for gene transfer and the production of transgenic poultry. Using gene-editing technologies such as CRISPR/Cas9, researchers have created multiple gene-edited chicken models, including immunoglobulin-knockout chickens, ovalbumin-knockout chickens, androgen receptor-knockout chickens, and chickens resistant to subgroup J avian leukosis virus [12,13,14,15]. These models have significantly advanced our understanding of avian gene function and facilitated the development of precise breeding strategies. Efficient transfection of PGCs is critical for in vitro gene expression studies and the generation of genetically modified PGCs. However, current lipofectamine-based transfection methods exhibit very low efficiency in avian PGCs, highlighting the urgent need to explore and optimize transfection strategies designed explicitly for PGCs. Further advancements in transfection efficiency will facilitate more precise genetic manipulation and enhance the utility of PGCs in avian functional genomics and genetic conservation.

In this study, we used electroporation to determine the optimal transfection conditions for PGCs in a feeder layer-free and serum-free medium. In addition, the study evaluated the effect on apoptosis and gene expression between different systems. The optimal electroporation models were used to deliver the enhanced green fluorescent protein (*EGFP*) gene to evaluate the gene knock efficacy.

## 2. Materials and Methods

### 2.1. PGC Acquisition and Cell Culture

The White Leghorn chicken embryos were utilized as the origin for PGCs isolation. The fertilized eggs were incubated until they reached 6.5 days of embryonic development. The gonads were extracted, and each was treated as a separate culture unit. The isolated gonads underwent three rinses with PBS before the application of 100 μL of 0.25% trypsin (25200056, Thermo Fisher Scientific, Waltham, MA, USA). Digestion proceeded for 7 min in a 37 °C incubator. An equivalent volume of serum-containing medium was introduced to halt the digestion. Following the addition of 350–400 μL of medium and a 3 h culture period, the suspended cells and supernatant were transferred to fresh wells for continued cultivation until cell line establishment. The PGCs medium composition was adapted from a previous study with slight modifications. The culture medium consisted of Avian Knockout Dulbecco’s Modified Eagle Medium (DMEM, 10829018, Thermo Fisher Scientific, Waltham, MA, USA) supplemented with 1 × B-27 (17504044, Thermo Fisher Scientific, Waltham, MA, USA), 1 × nonessential amino acids (11140050, Thermo Fisher Scientific, Waltham, MA, USA), 0.1 mM β-mercaptoethanol (ES-007-E, Sigma Aldrich, St Louis, MO, USA), 1 × nucleosides (12571063, Thermo Fisher Scientific, Waltham, MA, USA), 0.15 mM CaCl_2_ (C7902, Sigma Aldrich, St Louis, MO, USA), 0.01% sodium heparin (H3149, Sigma Aldrich, St Louis, MO, USA), 0.2% ovalbumin (S7951, Sigma Aldrich, St Louis, MO, USA), 5 μg/mL OT (C7786, Sigma Aldrich, St Louis, MO, USA), 4 ng/mL FGF2, 25 ng/mL Activin A (120-14, PeproTech, Rocky Hill, NJ, USA) and 1% KOSR (10828028, Thermo Fisher Scientific, Waltham, MA, USA) [16,17,18]. PGC cultures were maintained at 37 °C in an atmosphere containing 5% CO_2_.

### 2.2. Vector Construction

Editing plasmid construction. To insert the target gene into the chicken Z chromosome, the gRNA locus described in the previous literature was employed [19]. The primers for gRNA are in Supplementary Table S1. The primers were mixed in equal volumes at a working concentration of 10 μM, with 3.5 μL of each primer, and annealed to form double-stranded DNA. The double-stranded gRNA was cloned into the PX459 vector using Hifair^®^ Precision sgRNA Synthesis Kit (11355ES25, YEASEN, Shanghai, China) with the following components: PX459 plasmid 100 ng, double-stranded gRNA template 7 μL, 10 × FastDigest Buffer 1 μL, BbsI restriction enzyme 0.5 μL, 10 × T4 DNA Ligase Buffer 1 μL, and T4 ligase 0.5 μL. The ligation reaction was performed under the following program: 37 °C for 5 min, 22 °C for 5 min, repeated for 5 cycles.

Doner plasmid construction. An 800 bp sequence upstream and downstream of the gRNA target site in the chicken genome was amplified as the homologous arm (HA). The primers for amplifying HA are in Supplementary Table S2. The pB-PGK-EGFP plasmid was digested with XbaI and NotI to recover the backbone and the PGK-EGFP fragment. The donor plasmid containing HA was constructed using the Hieff Clone^®^ Plus Multi One Step Cloning Kit (10912ES10, YEASEN, Shanghai, China) following the reaction system described below: 2 × Hieff Clone^®^ MultiS Enzyme Premix: 10 µL; Vector backbone: 0.03 pmol; upstream HA: 0.03 pmol; downstream HA: 0.03 pmol; ddH₂O: to a total volume of 20 µL. The reaction mixture was incubated at 50 °C for 50 min.

### 2.3. Cell Transfection

PGCs were cultured in a feeder-free complete medium and seeded at 30–50% density in T75 culture flasks. The cells were incubated at 37 °C in a 5% CO_2_ incubator for 24 h to reach a 70–90% density before being harvested for transfection experiments.

Lipofectamine transfection. The cells were transferred into a centrifuge tube and centrifuged at 300× *g* for 5 min. The supernatant was removed, and the cell pellet was resuspended in 5 mL PBS for counting. Cell suspension was aliquoted into counting slides (145-0011, Bio-Rad, Hercules, CA, USA). Cell counting was performed using the TC-20TM Automated Cell Counter (Bio-Rad, Hercules, CA, USA), quantified via the count cells program. The PBS was removed after centrifuging again at 300× *g* for 5 min. Gradient pB-PGK-EGFP plasmids were combined with Lipofectamine™3000 (L3000015, Thermo Fisher Scientific, Waltham, MA, USA) in Opti-MEM (31985070, Thermo Fisher Scientific, Waltham, MA, USA), adhering to the manufacturer’s guidelines. This mixture was then introduced to 1 × 10^6^ PGCs and seeded into 6-well plates. Following a 6 h incubation period, the transfection mixture was removed and replaced with a fresh culture medium.

Transfection of Lonza electroporation system. The cells were transferred into a centrifuge tube and centrifuged at 300× *g* for 5 min. The supernatant was removed, and the cell pellet was resuspended in 5 mL PBS for counting. Cells were counted as described above. The PBS was removed after centrifuging again at 300× *g* for 5 min. A total of 1 × 10^6^ PGCs were resuspended in 100 μL of Entranster™-E electroporation buffer (98668-20, Engreen, Beijing, China) mixed with varying amounts of pB-PGK-EGFP plasmid. The transfection mixture was then transferred into an electroporation cuvette and subjected to electroporation using the Lonza AAD-1001S Nucleofector^®^ system (Lonza, Baden-wurttemberg, Germany). For miRNA mimic and inhibitor transfection, the transfection mixture consisted of 1 × 10^6^ PGCs, 100 μL of Entranster™-E electroporation buffer, and a final culture concentration of 200 nM of mimic (miR1CM001, Ribobio, Guangzhou, China) or inhibitor (miR2CM001, Ribobio, Guangzhou, China). The components were thoroughly mixed and electroporated using the B16 program. For Z chromosome insertion transfection, the transfection system included 1 × 10^6^ PGCs, 100 μL of Entranster™-E buffer, 2 μg of editing plasmid, and 3 μg of donor plasmid. The components were mixed well and subjected to electroporation using the B16 program.

Transfection of Thermo electroporation system. The cells were transferred into a centrifuge tube and centrifuged at 300× *g* for 5 min. The supernatant was removed, and the cell pellet was resuspended in 5 mL PBS for counting. Cells were counted as described above. After another centrifugation at 300× *g* for 5 min, the PBS was removed. A total of 1 × 10^6^ PGCs were resuspended in 100 μL of Neon™ NxT Resuspension Buffer R (N10096, Thermo Fisher Scientific, Waltham, MA, USA) mixed with varying amounts of pB-PGK-EGFP plasmid. The transfection mixture was transferred into an electroporation cuvette and electroporated using the Thermo Neon™ NxT electroporation system (Thermo Fisher Scientific, Waltham, MA, USA).

Post electroporation, 1 × 10^6^ PGCs were seeded into 6-well plates and maintained in complete medium at 37 °C.

### 2.4. Flow Cytometry Assay

Transfection analysis. Following a 48 h transfection period, PGCs were harvested 300× *g* for 5 min and washed twice with PBS. Cells were transferred to BD tubes (Becton Dickinson, Franklin Lakes, NJ, USA) for detection of the fluorescent signal by flow cytometry using the BD FACS CANTO II (Becton Dickinson, Franklin Lakes, NJ, USA). EGFP fluorescence was excited by laser with wavelength 488 nm and detected in filter 530 nm. Data were then analyzed with the FlowJo software (version 10.8.1, Becton Dickinson, Franklin Lakes, NJ, USA).

Cell apoptosis analysis. Following a 24 h transfection period (the vector in this assay is pcDNA3.0), cells were harvested 300× *g* for 5 min and washed twice with PBS. Cell mortality was evaluated using the PE Annexin V Apoptosis Detection Kit (BD 559763, Biosciences, San Jose, CA, USA), adhering strictly to the manufacturer’s guidelines. PGCs were resuspended in 100 μL of binding buffer containing 5 μL Annexin V-PE and 5 μL 7-ADD for 30 min in darkness at room temperature. The percentage of apoptotic cells was then detected by flow cytometry. EGFP-7-ADD fluorescence was excited by a laser with wavelength 488 nm and detected in filter 530 nm. PE Annexin V fluorescence was excited by a laser with wavelength 560 nm and detected in filter 580 nm. Data were then analyzed with the FlowJo software (version 10.8.1, Becton Dickinson, Franklin Lakes, NJ, USA).

### 2.5. RT-qPCR

Following a 48 h transfection period, cells were collected at 300× *g* for 5 min and washed twice with PBS. Total RNA was isolated from PGCs employing TRIzol Reagent (T9424, Sigma Aldrich, St Louis, MO, USA). Subsequently, the RNA was reverse transcribed to cDNA using the Fastking RT kit (KR116-02, Tiangen, Beijing, China) and the miDETECT A Track miRNA qRT-PCR Starter Kit (C10712-2, Ribobio, Guangzhou, China), adhering to the manufacturer’s guidelines. Supplementary Table S3 provides a comprehensive list of the specific quantitative primers utilized. The RT-qPCR was performed on a CFX96 Touch Deep Well Real-Time PCR Detection System (CFX96, Bio-rad, Hercules, CA, USA) using a reaction mixture containing cDNA, 2 × SuperReal PreMix Plus SYBR Green (FP205, Tiangen, Beijing, China), and the appropriate forward and reverse primers.

### 2.6. Genotyping and Sequencing

The extraction of genomic DNA was performed using the HiPure Universal DNA Kit (D3018-03, Magen, Guangzhou, China). For genotype identification, polymerase chain reaction (PCR) was conducted using the primers specified in Supplementary Table S4. Gel electrophoresis was employed to analyze the PCR products, followed by sequencing the insertion site at Tianyihuiyuan Company (Beijing, China) for further analysis.

### 2.7. Statistical Analysis

Experiments data were reported as the mean ± SEM. A *t*-test was used to analyze the significant difference between the treatment and control groups. Multiple comparisons were assessed using one-way ANOVA with Dunnett’s post hoc test using SPSS 24.0 software. For all statistical analyses, *: *p* ≤ 0.05, **: *p* ≤ 0.01, ***: *p* ≤ 0.001, n.s.: not significant.

## 3. Results

### 3.1. Electroporation Efficiency of PGC Is Better than That of Lipofectamine

**Figure 1:**
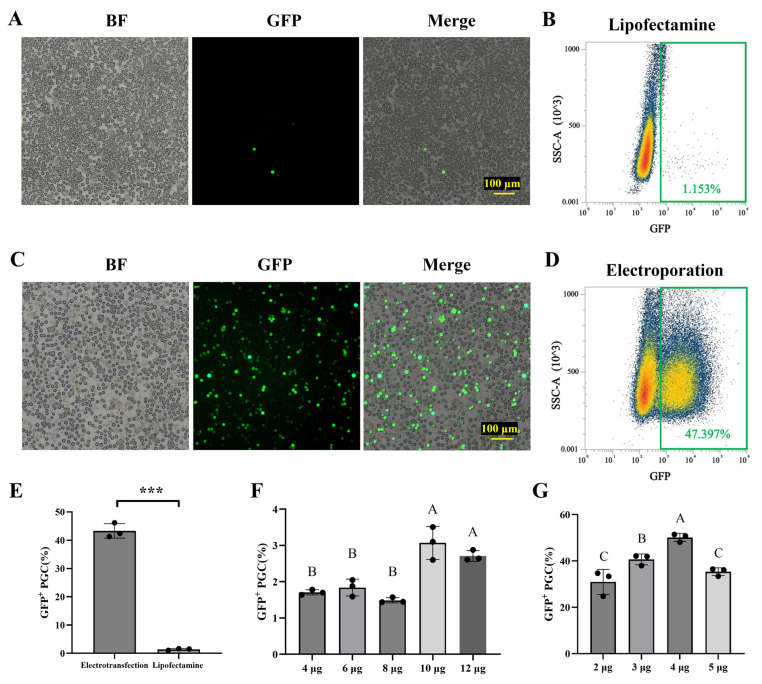
Comparison and optimization of electroporation and lipofectamine transfection efficiency. pB-PGK-EGFP expression vector was transfected into primordial germ cells (PGCs) utilizing electroporation (**A**,**B**) or lipofectamine (**C**,**D**). Scale bars, 100 μm. (**E**) The transfection efficiency of different transfection methods was analyzed via flow cytometry. Data are presented as mean ± standard error, *n* = 3. ***: *p* < 0.001. (**F**) The transfection efficiency of varying quantities of a vector by lipofectamine. (**G**) The transfection efficiency of varying quantities of vector by electroporation. Distinct alphabetical designations indicate statistically significant differences between groups at *p* < 0.01.

First, the study compared the efficiency of lipofectamine and electroporation on PGCs. The results show that a low proportion of EGFP-positive cells (1.38 ± 0.34%) were observed after 48 h transfection by using Lipofectamine ™ 3000 and 4 μg of plasmid (Figure 1A,B,E). In contrast, when utilizing the electroporation system of Thermo, the proportion of positive PGCs reached 43.29 ± 2.56% at a condition of 1100 V 40 ms 1 plus (Figure 1C–E). The quantity of vector used for lipofectamine and electroporation was optimized. The proportion of EGFP-positive PGCs transfected using lipofectamine did not exhibit an increase as the plasmid dosage increased (Figure 1F). While the positive rate increased in electroporation, reaching a maximum (50.10 ± 1.75%) when using 4 μg (1 pmol) with 1 × 10^6^ Cells, after which the positive rate decreased with further increases in plasmid dosage (Figure 1G). Unless otherwise specified, this vector dosage was used for subsequent experiments. These results demonstrated that the electroporation efficiency in PGCs without a feeder layer was better than that of lipofectamine, and the optimal concentration for 1 × 10^6^ cells was 4 μg (1 pmol).

### 3.2. Optimization of Electroporation Efficiency

**Figure 2:**
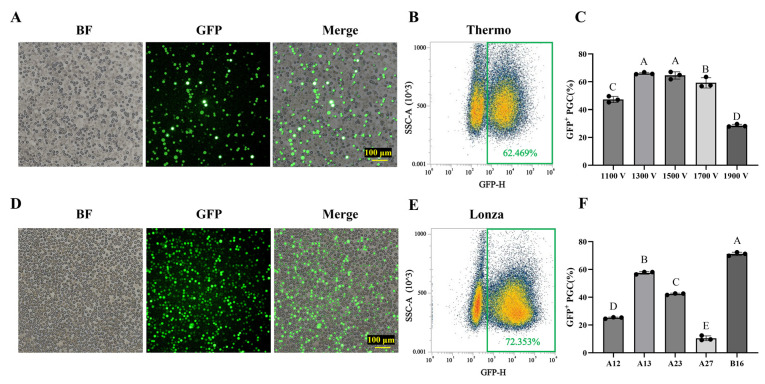
Optimization of transfection protocols for various electroporation systems. (**A**,**B**) Transfection efficiency of PGCs based on the Thermo system. PGCs from White Leghorn were transfected with the pB-PGK-EGFP expression vector using the Thermo electroporation apparatus (parameters: 1100 V, 40 ms, single pulse). Scale bars represent 100 μm. (**C**) The efficacy of various transfection procedures on the Thermo system was assessed via flow cytometry. Results are expressed as mean ± standard error, *n* = 3. (**D**,**E**) Transfection efficiency of PGCs based on the Thermo system. White Leghorn PGCs underwent transfection with the pB-PGK-EGFP expression vector using the Lonza electroporation system (program: B16). Scale bars indicate 100 μm. (**F**) The efficacy of various transfection procedures on the Lonza system was assessed via flow cytometry. Data are presented as mean ± standard error, *n* = 3. Distinct alphabetical designations indicate statistically significant differences between groups at *p* < 0.01.

The efficiency of electroporation was further optimized. The results presented in Figure 2A–C and in Figure S1 demonstrate that the EGFP-positive rate of PGCs increased with increasing voltage, reaching a maximum value (66.03 ± 0.75%) when utilizing 1300 V. As the voltage continued to increase, the positive rate decreased significantly. Similar experiments were conducted on the Lonza system. On this system, the transfection efficiency varied considerably among different programs, with the B16 program exhibiting the highest efficiency at 71.13 ± 1.26% (Figure 2D–F and Figure S2). In conclusion, both electroporation systems achieved transfection efficiencies exceeding 60% after optimization.

### 3.3. Effects of Transfection on Apoptosis and Gene Expression

**Figure 3:**
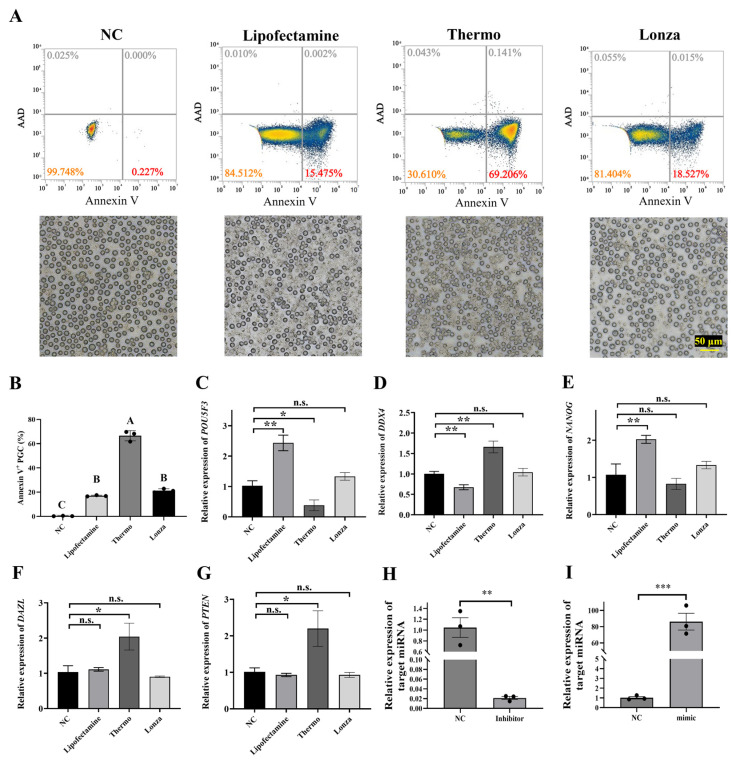
Impact of different transfection modalities on PGCs apoptosis and gene expression. (**A**,**B**) Apoptotic analysis of PGCs subjected to various transfection methodologies. PGCs underwent transfection with the pCDNA3.0 vector using different approaches for a 24 h duration. Flow cytometry was employed to detect Annexin V (GFP) and 7-AAD (RFP) protein staining. Quantitative data are expressed as mean ± standard error, *n* = 3. Distinct alphabetical designations indicate statistically significant differences between groups at *p* < 0.01. (**C**–**G**) Influence of different transfection modalities with the pB-PGK-EGFP vector on mRNA expression levels of *DAZL*, *DDX4*, *NANOG*, *POU5F3*, and *PTEN* in PGCs. Following transfection of the pB-PGK-EGFP expression vectors into PGCs via multiple methods, RT-qPCR analysis was conducted to assess the expression of genes after 48 h. Results are presented as mean ± standard error, *n* = 3. n.s.: not significant, *: *p* < 0.05, **: *p* < 0.01. (**H**) Inhibitors of miRNA were delivered into PGCs through using the Lonza electroporation system to suppress target miRNA expression. RT-qPCR quantified target miRNA levels 48 h post-transfection. Results are depicted as mean ± standard error. *n* = 3, **: *p* < 0.01. (**I**) Mimics of miRNA were electroporated into PGCs to enhance target miRNA expression. Results are depicted as mean ± standard error. *n* = 3, ***: *p* < 0.001.

Different transfection methods can cause varying levels of cellular damage; the study has assessed their impact on apoptosis. The proportion of PGCs expressing Annexin V was 16.99 ± 0.52%, 21.27 ± 1.54%, and 66.57 ± 4.21% after 24 h in lipofectamine, the Lonza system, and the Thermo system, respectively (Figure 3A,B). This research examined the expression of PGC marker genes and pluripotency genes 48 h post-transfection to account for the potential impact of different transfection methods on the production of PGCs. The results indicated that the use of the Lonza system did not affect the expression of PGC marker genes *DAZL* and *DDX4*, as well as pluripotency genes *NANOG*, *POU5F3*, and *PTEN* (Figure 3C–F). However, electro-transfer using the Thermo system led to a significant increase in the expression of *DAZL*, *DDX4*, and *PTEN*, and a significant decrease in *POU5F3* expression. In contrast, the results from lipofectamine indicated a significantly increased expression of *NANOG* and *POU5F3*, while the expression of *DDX4* significantly decreased after 48 h of transfection. Furthermore, the study evaluated the feasibility of delivering RNA using the Lonza system. Inhibitors and mimics of miR-17-5p were delivered to PGC to confirm the inhibition and overexpression efficiencies. The results demonstrated that the relative expression of miRNAs in PGCs decreased 0.02-fold and increased 86-fold, respectively (Figure 3H,I). These findings suggest that the Lonza system can ensure optimal transfection efficiency while maintaining cell viability and PGC function.

### 3.4. Stable Transfection of PGCs with Lonza System

**Figure 4:**
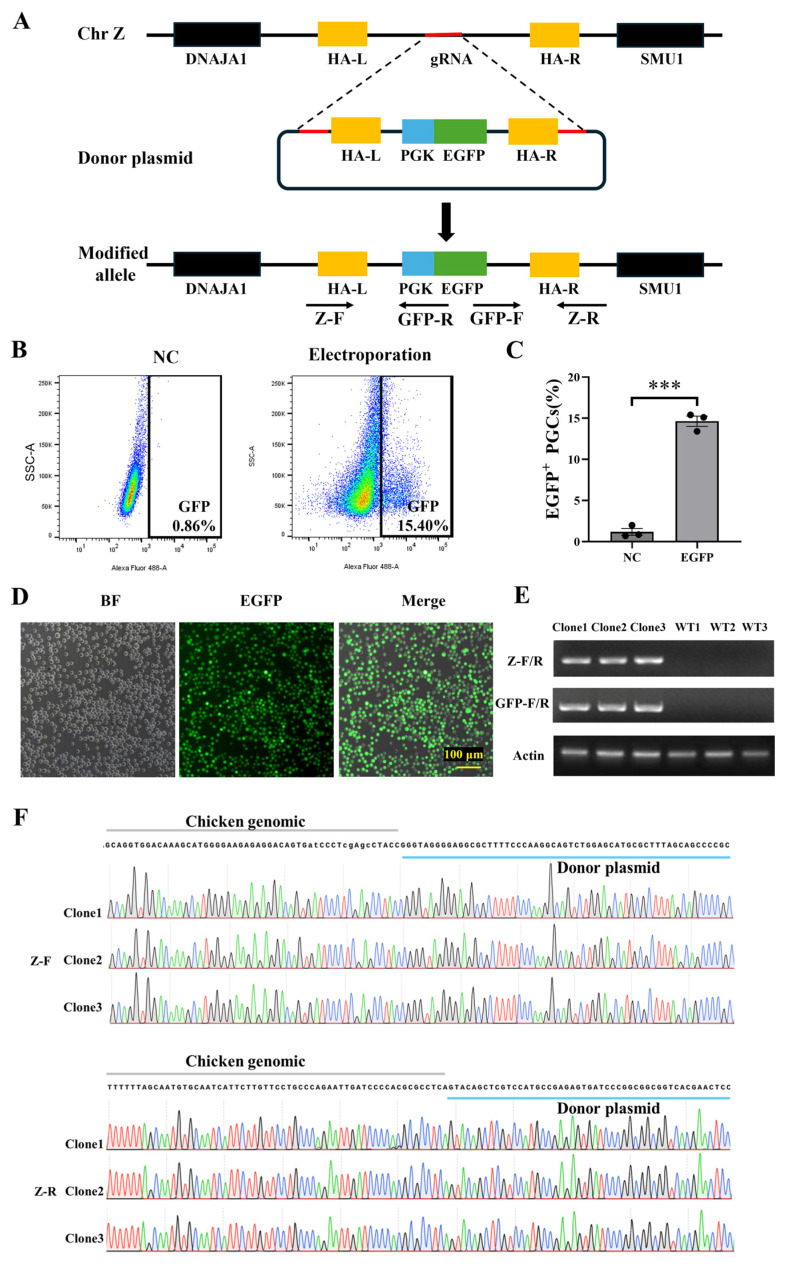
PGCs model construction via electroporation. (**A**) The schematic depicts gene insertion into the Z chromosome’s intergenic region in PGCs. Enhanced green fluorescent protein (*EGFP*) expression from the Z chromosome was facilitated by introducing a CRISPR/Cas9 vector targeting the intergenic region, alongside a donor plasmid with gRNA recognition sites. Orange, blue, green, and red denote HA, PGK promoter, EGFP, and gRNA sequences, respectively. (**B**,**C**) The efficiency and statistics of targeted Z chromosome insertion were assessed by flow cytometry. PGCs were electroporated with double plasmids, cultured for 7 days, then sorted and enumerated via flow cytometry. Results are presented as mean ± standard error, *n* = 3. ***: *p* < 0.001. (**D**) Fluorescence observation of sorted PGCs. PGSs continued to express EGFP fluorescence in subsequent cultures. (**E**,**F**) PCR and subsequent sequencing of PCR products were employed to analyze genomic DNA for targeted gene insertion in PGCs.

The study also assessed the effect of constructing gene knock-in PGC lines based on this electroporation system. Using the CRISPR/Cas9 system, we targeted a specific locus on the Z chromosome to establish EGFP-stably expressing PGCs. This locus has been previously reported as a safe harbor site [19]. HA were introduced, flanking the target site to improve the efficiency of homologous recombination (Figure 4A). After the targeted sequence was integrated into the genome, the PGCs continuously expressed EGFP through the *PGK* promoter, while the fluorescence gradually diminished in cells without the knock-in. Knock-in efficiency was evaluated by flow sorting at 7 days after transfection. (Figure 4B,C), and the EGFP positive cells were 14.63 ± 1.07%. The PGCs obtained by flow sorting were maintained in culture with persistent fluorescence expression (Figure 4D). The PCR and sequencing results indicated the accuracy of site insertion (Figure 4E,F). These results reveal that the production of gene-edited PGCs using the Lonza system was feasible and efficient.

## 4. Discussion

In this study, we aimed to explore an efficient transfection method for PGCs in poultry. Under feeder-free and serum-free culture conditions, the lipofectamine-mediated transfection efficiency of chicken PGCs was very low, which is consistent with previous findings [2]. To address this limitation, we optimized the electroporation-based transfection method for PGCs to establish an efficient transfection system. First, we investigated the impact of different plasmid dosages on electroporation efficiency. We also compared the transfection efficiency of two electroporation systems by optimizing various parameter gradients for each system. The results indicated that the Lonza electroporation system achieved the highest transfection efficiency, with a peak transient efficiency of 71.13 ± 1.26% (Figure 2). Moreover, this optimized system did not disrupt the expression of PGC marker genes or pluripotency genes, resulting in reduced apoptosis. This method introduced miRNA mimics and inhibitors into PGCs, leading to an increase in target miRNA expression by 86-fold and a decrease by 0.02-fold, respectively. We also successfully generated Z chromosome-integrated gene-edited PGCs via CRISPR/Cas9 and homologous recombination (HR) repair, achieving a 7-day positive rate of 14.63 ± 1.07%, the highest efficiency reported so far.

Lipofectamine-based transfection is one of the most commonly used methods for cell transfection [20]. However, the efficiency of lipofectamine-mediated transfection is notably low in suspension primary cells, particularly in chicken PGCs [2]. Macdonald’s study utilized the piggyBac and Tol2 transposon systems with Lipofectamine for PGC delivery, achieving a transfection efficiency of 10.5% [21]. The composition between feeder-containing and feeder-free culture systems is quite different, which may impact the efficiency of lipofectamine-based transfection in PGCs. Several researchers have attempted to optimize and explore methods to enhance the transfection efficiency of chicken PGCs. In the study, conducted by Tenkai et al., the removal of heparin sodium increased the efficiency of PGC transfection with Lipofectamine™ 3000 from less than 1% to 16% [22]. The serum content in the culture medium is another important factor influencing lipofectamine-mediated transfection. In a feeder-free culture system without heparin sodium, the presence of serum (1%) increased the transfection efficiency of Lipofectamine™ 2000 (11668019, Thermo Fisher Scientific, Waltham, MA, USA) by threefold compared to serum-free conditions, achieving a maximum efficiency of 64% [22]. However, in the present study, removing heparin sodium from the culture system did not enhance lipofectamine transfection efficiency. This difference may be due to the different culture systems. Given the complexity and variability of feeder-free PGC culture systems, lipofectamine-based transfection remains limited in its applicability to PGCs.

Electroporation is commonly used for “refractory cells” due to its relatively high efficiency in delivering molecules into cells. When cells are exposed to an electric field of sufficient intensity, the permeability of the plasma membrane increases, allowing previously impermeable molecules to enter the cells through reversible electroporation [4,5]. This method is theoretically applicable to all cell types, including stem cells. However, the electroporation conditions vary significantly between different cell types, so optimizing the vector and the electroporation program is important to achieve the highest transfection efficiency [5,23]. Altgilbers et al. used a Thermo electroporation system to deliver the Sleeping Beauty transposon system to PGCs (1300 V, 10 ms, 4 pulse) and achieved a transfection efficiency of about 65%, which was comparable to the results in this study [24]. The Lonza electroporation system with the B16 program used in this study achieved a significantly higher transfection efficiency of 71.13 ± 1.26% (Figure 2). The Lonza system and program were initially utilized to transfect human embryonic stem cells (hES). Its transfection efficiency in trypsin-dissociated H9 hES cells was more than 70% [25]. Later, this system was applied to transfect pluripotent stem cells and hematopoietic stem cells. However, no studies reported its use in PGCs. Here, we prove that the Lonza system and program work well for feeder-free PGC transfection. It also suggests that it may be suitable for other germ cells, such as spermatogonial stem cells, while further validation is needed.

Despite its advantages in transfection efficiency, electroporation’s major limitation is the high cell mortality rate, which restricts its broader applicability. In contrast, the Lonza system uses cell-specific buffers and programs to optimize transfection to reduces cell death [26]. Compared to the Thermo system, the Lonza shows higher cell survival and better cellular status at 48 h post-transfection. This was also confirmed by apoptosis assays at 24 h after transfection. The Thermo system showed a significantly higher ratio of early apoptosis than the Lonza. Subsequent analysis of PGC marker and pluripotency gene expression further corroborated this observation. PGC markers, including *DDX4* and *DAZL*, are crucial for differentiating PGCs and developing the germline [27,28]. Meanwhile, *PTEN*, *NANOG*, and *POU5F3* are essential in maintaining pluripotency and self-renewal [20,29,30]. Both Lipofectamine-based transfection and the Thermo electroporation system led to aberrant expression of these genes, while the Lonza system successfully preserved their normal expression profiles.

Genome editing in chicken PGCs was successfully performed using an optimized electroporation-based transfection protocol. Under these optimized electroporation conditions, the co-transfection of the CRISPR/Cas9 vector and donor vector into PGCs achieved a site-specific insertion efficiency of 14.63 ± 1.07% (Figure 4C). In comparison, previous studies by Tenkai et al. reported an insertion efficiency of less than 1% for EGFP knock-in at the *ACTB* locus using Lipofectamine™ 2000 and HR [22]. Similarly, Xie et al. achieved editing efficiencies of 6.25% and 0% for *DAZL* and *POU5F3* loci knock-in with mCherry in PGCs, respectively, using Lipofectamine™ 3000 and HR strategies [31]. Though the integration efficiency varied depending on the targeted knock-in site, the HR-mediated knock-in efficiency achieved through electroporation consistently surpassed that of Lipofectamine-based transfection. Notably, the homology-mediated end joining (HMEJ) strategy further improved the import efficiency by 2- to 4-fold compared with the HR strategy, underlining the importance of selecting an appropriate knockout strategy to improve editing efficiency [22]. Although the culture system less affects electroporation, the selection of a knock-in strategy and site is an important step in optimizing electroporation efficiency.

## 5. Conclusions

This study demonstrates the efficacy of Lonza electroporation in achieving high transfection efficiency for PGCs under feeder-free conditions. The versatile method enables miRNA manipulation experiments and genome editing through CRISPR/Cas9 technology. These findings lay the groundwork for advancing in vitro PGCs research and producing genetically edited avian species.

## Data Availability

The original contributions presented in this study are included in the article/Supplementary Materials. Further inquiries can be directed to the corresponding author.

## Supplementary Materials

The following supporting information can be downloaded at: https://www.mdpi.com/article/10.3390/ani15040590/s1, Figure S1: Transfection effect of different procedures in the Thermo system; Figure S2: Transfection effect of different procedures in the Lonza system; Table S1: The primers for gRNA; Table S2: The primers for amplifying HA; Table S3: The primers for RT-qPCR; Table S4: The primers for PCR.
