# Supplementary material for: Achieving Optimal Transfection Conditions in Chicken Primordial Germ Cells Under Feeder- and Serum-Free Medium
Source: Animals (Basel). 2025 Feb 18;15(4):590. doi: 10.3390/ani15040590 (PMC11851890; doi:10.3390/ani15040590)
Supplement: Supplementary file 1 [file animals-15-00590-s001.zip › Supplementary Figure S1-S2.pdf]

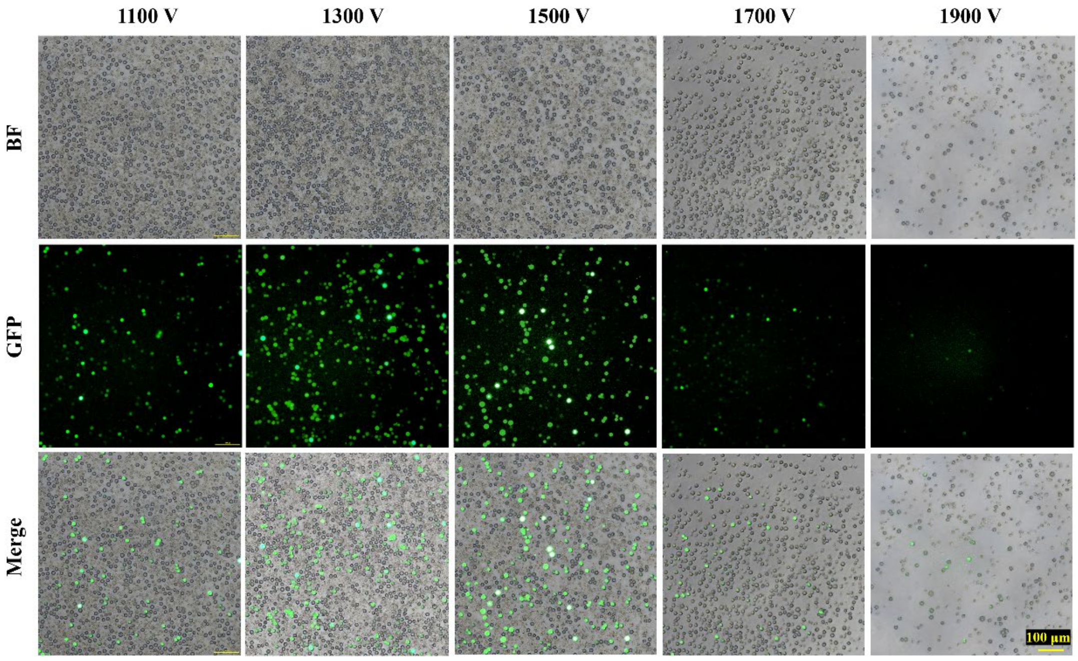

Supplementary Figure S1. Transfection effect of different procedures in the Thermo system. A total of 1 × 10^6 PGCs were resuspended in 100 μL of Neon™ NxT Resuspension Buffer R (N10096, Thermo Fisher Scientific, Waltham, MA, USA) mixed with 4 μg of pB-PGK-EGFP plasmid. The transfection mixture was transferred into an electroporation cuvette and electroporated with different program using the Thermo Neon™ NxT electroporation system.

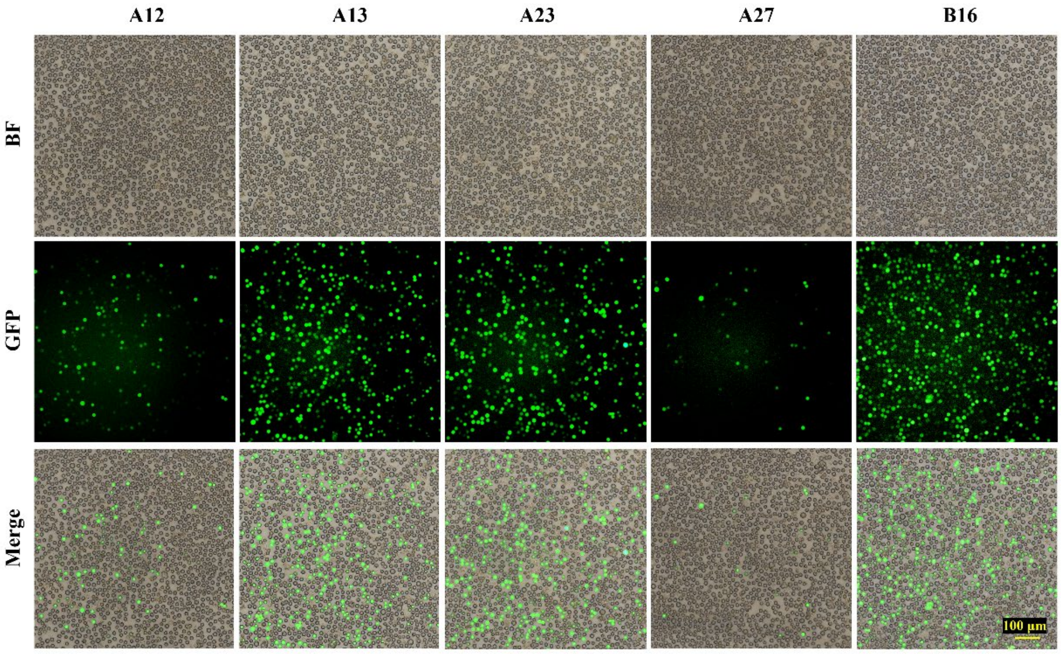

Supplementary Figure S2. Transfection effect of different procedures in the Lonza system. A total of 1 × 10^6 PGCs were resuspended in 100 μL of Entranster™-E electroporation buffer (98668-20, Engreen, Beijing, China) mixed with 4 μg of pB-PGK-EGFP plasmid. The transfection mixture was then transferred into an electroporation cuvette and subjected to electroporation with different program using the Lonza AAD-1001S Nucleofector® system (Lonza system).
